# Optimized Fertilization Enhances Wheat (*Triticum aestivum* L.) Yield and Quality in Ningxia Irrigated Silty Soil: Physio-Ecological Mechanisms

**DOI:** 10.3390/plants15121902

**Published:** 2026-06-19

**Authors:** Yuanyuan Hu, Qian Zheng, Pan Xie, Jinrong Yang, Wei Lin

**Affiliations:** 1 Research Center for Crop Biotechnology Breeding and Smart Cultivation in Southern Xinjiang, College of Modern Agriculture, Kashi University, Kashi 844000, China; 13037980321@163.com (Y.H.); zhengqian901122@163.com (Q.Z.); panxie126@sina.com (P.X.); love_jsu@163.com (J.Y.); 2 Institute of Urban Agriculture, Chinese Academy of Agricultural Sciences, Chengdu 610213, China

**Keywords:** wheat, Ningxia dry farming area, recommended fertilization, soil nutrients, yield

## Abstract

Identifying soil nutrient limiting factors and fertilization effects in the irrigated silty soil region of Ningxia is key to improving wheat (*Triticum aestivum* L.) quality and yield. A field experiment was conducted with five treatments: conventional fertilization (TF), recommended fertilization (RF), nitrogen deficiency (RF-N), phosphorus deficiency (RF-P), and potassium deficiency (RF-K). The results showed that under RF, soil nutrients remained at relatively high levels, with no significant differences compared with TF. In contrast, RF-N significantly reduced soil mineral nitrogen, total nitrogen, and organic matter compared with TF, and inhibited plant growth, photosynthesis, and plant accumulation of nitrogen, phosphorus, and potassium. Wheat yields under RF and RF-K showed no significant differences from those under TF, whereas RF-N and RF-P significantly reduced yields by 42.68% and 22.69%, respectively, relative to RF, mainly due to decreases in spike length and grain number per spike. The increase in yield was associated with synergistic increases in grain number per spike, spike number per hectare, and spike length. Yield components were significantly positively correlated with soil organic matter, total phosphorus, and mineral nitrogen, with soil total phosphorus identified as the environmental factor most strongly associated with wheat yield. Grain protein content was significantly positively correlated with soil mineral nitrogen, while starch content was significantly negatively correlated, indicating that mineral nitrogen is a key factor regulating grain quality. In summary, nitrogen fertilizer is the primary limiting factor in this region. Applying nitrogen, phosphorus, and potassium together synergistically enhances wheat yield by increasing soil total phosphorus levels and improves grain quality by regulating soil mineral nitrogen. Thus, this combined fertilization strategy provides a foundation for precise nutrient management and the simultaneous improvement of both yield and quality.

## 1. Introduction

The Ningxia Yellow River Diversion Irrigation Area is a crucial grain production base in the arid region of Northwest China. The unique irrigated silty soil in this region, formed by over two thousand years of Yellow River irrigation and anthropogenic cultivation [1], occupies approximately 34% of the cultivated land and contributes more than 70% of the grain output, thus playing a vital strategic role [2]. However, the sustainable productivity of this “granary beyond the Great Wall” faces severe challenges. On the one hand, the region is characterized by an arid climate with annual precipitation of only 200–400 mm, making agricultural production highly dependent on Yellow River irrigation, while water resource constraints are becoming increasingly stringent. On the other hand, the extensive management model of “high water and fertilizer input for high yield” developed over a long period, particularly the excessive application of nitrogen fertilizer and imbalanced phosphorus and potassium ratios, has led to a decline in fertilizer use efficiency, aggravated environmental pollution, and soil quality degradation [3], posing a serious threat to the long-term health of the regional agricultural system. Therefore, under the dual strategic goals of ensuring national food security and promoting green agricultural transformation, how to achieve “fertilizer reduction with efficiency enhancement” in wheat (*Triticum aestivum* L.) production on the irrigated silty soils of Ningxia has become an urgent practical and scientific issue.

The long-term irrigation and sedimentation of irrigated silty soil have endowed it with a considerable total nutrient pool. However, its elevated pH and high calcium carbonate content lead to pronounced phosphorus fixation, which sharply diminishes phosphorus availability and results in a well-recognized “abundant reserve but low availability” dilemma. The phosphorus fixation capacity of Ningxia’s irrigated silty soil is high, with more than 60% of added phosphate being converted into plant-unavailable forms [4]. The calcium carbonate concentration in this soil varies between 45 and 75 g kg^−1^ [5]. Consequently, under conventional fertilization regimes, the phosphorus-use efficiency in this region remains typically low, ranging from 10% to 20% [6]. This soil chemical characteristic often renders conventional fertilization recommendations based on total soil nutrient content ineffective. Meanwhile, due to a lack of scientific guidance, current farmer fertilization practices commonly suffer from excessive nitrogen application and low phosphorus and potassium use efficiency. How to balance soil nutrient supply and demand to achieve fertilizer reduction and efficiency enhancement has become a focus of academic attention. Early studies showed that excessive nitrogen application not only reduces nitrogen use efficiency but may also inhibit crop uptake of other nutrients [7,8], whereas phosphorus and potassium deficiencies directly limit crop growth and yield formation [9,10]). With further research, the critical importance of balanced nutrient supply became increasingly evident. Studies on various crops and soils have confirmed that the rational combined application of nitrogen, phosphorus, and potassium produces significant synergistic effects, ultimately achieving coordinated improvements in crop yield and resource use efficiency by optimizing root architecture, enhancing photosynthetic capacity, and promoting the translocation of photosynthates to grains [11].

Nevertheless, current research suffers from several important shortcomings. First, the majority of studies have concentrated on common soil types, including fluvo-aquic soil and red soil, while systematic studies of the distinctive “anthropogenic soil”—the irrigated silty soil of Northwest China—remain markedly insufficient. Second, existing work has primarily focused on final crop yield, offering limited insight into the sequential mechanisms through which fertilizer reduction affects key soil properties, subsequently influencing plant physiological processes and ultimately determining yield components and grain quality. To address these gaps, the present study quantitatively defines the “fertilizer reduction threshold” as the minimal application rates of nitrogen (N), phosphorus (P_2_O_5_), and potassium (K_2_O) required to sustain grain yield and quality comparable to those achieved under conventional farmer practice (TF), while concurrently improving nutrient-use efficiency. Based on preliminary experiments and regional fertilizer recommendations, the established reduction thresholds are: N reduced from 270 to 215 kg ha^−1^ (a 20.4% decrease), P_2_O_5_ reduced from 120 to 95 kg ha^−1^ (20.8% decrease), and K_2_O reduced from 75 to 60 kg ha^−1^ (20.0% decrease). Consequently, a systematic field experiment was conducted on the irrigated silty soil of Ningxia. This study was designed not only to evaluate the impacts of reducing individual nutrients but also to deeply explore the limitation hierarchy and synergistic mechanisms among nitrogen, phosphorus, and potassium. Accordingly, this research seeks to answer three key questions: (1) Which nutrient is the primary limiting factor for wheat yield and quality in irrigated silty soil? (2) How does reduced fertilization affect wheat growth and yield formation by altering soil nutrient availability and plant physiological processes? (3) What are the core soil indicators that drive the formation of final yield and quality?

## 2. Results

### 2.1. Effects of Different Fertilization Treatments on Topsoil Nutrients

Among all treatments, the recommended fertilization (RF) treatment had the highest contents of soil available potassium, available phosphorus, mineral nitrogen, total nitrogen, total phosphorus, and organic matter, and these indicators did not differ significantly from those under conventional fertilization (TF) (Table 1). In contrast, the nutrient deficiency treatments significantly decreased certain soil nutrient indicators (*p* < 0.01). Among these, the nitrogen deficiency treatment (RF-N) showed the most pronounced decreases. Compared with TF, soil mineral nitrogen, total nitrogen, and organic matter contents under RF-N decreased by 27.34%, 18.87%, and 24.88%, respectively. The phosphorus deficiency (RF-P) treatment also significantly reduced soil mineral nitrogen and organic matter contents (*p* < 0.01). In contrast, the potassium deficiency (RF-K) treatment exerted minimal effects on soil nitrogen and organic matter contents.

### 2.2. Effects of Different Fertilization Treatments on Wheat Growth and Photosynthetic Parameters

#### 2.2.1. Effects on Wheat Growth

**Figure 1:**
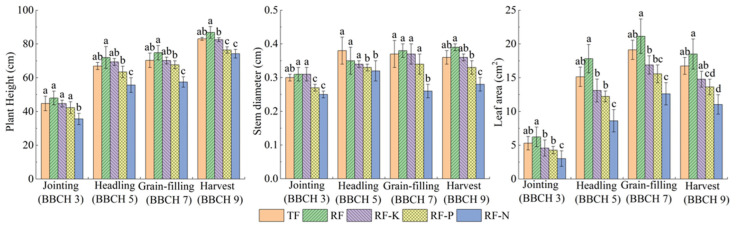
Changes in wheat (*Triticum aestivum* L.) plant growth under different fertilization treatments. Different letters indicate significant differences among treatments (*p* < 0.05).

Across all treatments, wheat plant height and stem diameter increased as the growth stage progressed, reaching their maximum at harvest (BBCH 9). Leaf area, however, attained its peak earlier, during the grain-filling stage (BBCH 7) (Figure 1). The RF, RF-K, and RF-P treatments did not significantly alter plant morphological parameters relative to the TF treatment. Starting from the jointing stage, plant height in the RF-N treatment was markedly lower than that in the TF treatment. At harvest, plant height under RF-N was 10.59% and 14.49% lower than that under TF and RF treatments, respectively. From jointing to the heading stage, stem diameter in the RF-N treatment was the smallest among all treatments, with a significant reduction of 15.79% compared to TF; thereafter, stem diameter under RF-N continued to decline. Leaf area in the RF-N treatment remained the lowest throughout the entire growth period.

#### 2.2.2. Effects on Wheat Photosynthetic Parameters

**Figure 2:**
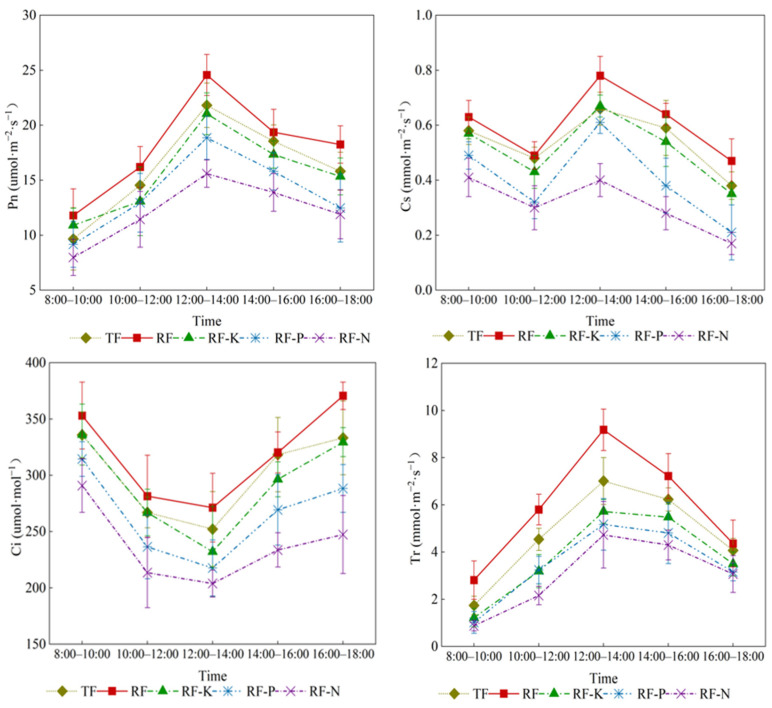
Changes in photosynthetic characteristics of wheat (*Triticum aestivum* L.) flag leaves under different fertilization treatments.

Fertilization treatments significantly influenced the photosynthetic characteristics of wheat flag leaves during the filling stage (BBCH 7) (*p* < 0.05) (Figure 2). Overall, the RF treatment showed the highest photosynthetic performance, the RF-K treatment showed similar effects to the TF treatment, whereas both RF-N and RF-P markedly suppressed photosynthesis, with nitrogen deficiency causing the strongest reduction. In terms of diurnal variation, the net photosynthetic rate (Pn) and transpiration rate (Tr) of flag leaves across all treatments first increased and then decreased, with peak values observed between 12:00 and 14:00. After 12:00, Pn under RF-N was significantly lower than that under RF and TF. Stomatal conductance (Gs) first declined from 8:00 to 10:00 and from 10:00 to 12:00, then gradually increased, peaking at 12:00–14:00, and subsequently decreased continuously. Gs under TF remained relatively high throughout the day, while Gs under RF-N was significantly lower than under RF. Intercellular CO_2_ concentration (Ci) initially decreased and then increased, reaching its lowest point at 12:00–14:00, with Ci under RF-N being significantly lower for most of the day.

#### 2.2.3. Effects of Different Fertilization Treatments on Nutrient Uptake of Wheat

**Figure 3:**
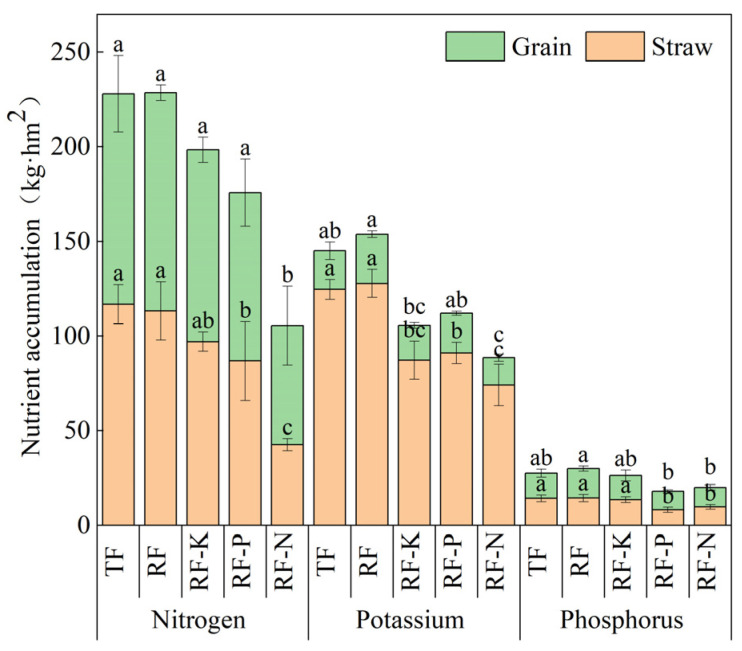
Changes in nutrient uptake in various organs of spring wheat (*Triticum aestivum* L.) under different fertilization treatments. Different letters indicate significant differences among treatments (*p* < 0.05).

As shown in Figure 3, compared with TF, the RF treatment showed no significant differences in nitrogen, phosphorus, or potassium in either the vegetative organs or the grains of wheat. In contrast, the RF-N treatment significantly reduced nitrogen and phosphorus accumulation in all parts of the wheat plant (*p* < 0.05). Potassium was primarily stored in the vegetative organs. Compared with the TF treatment, the RF-K, RF-P, and RF-N treatments all caused significantly decreases in potassium accumulation in the vegetative organs (*p* < 0.05), with reductions of 30.06%, 27.07%, and 40.54%, respectively, and the greatest decrease occurred under nitrogen deficiency. Furthermore, nitrogen deficiency also significantly reduced potassium accumulation in grains (*p* < 0.05).

### 2.3. Effects of Different Fertilization Treatments on Wheat Yield and Yield Components

Relative to TF treatment, wheat yield under the RF and RF-K treatments remained statistically unchanged (Table 2). In the RF-N treatment, both spike length and grain number per spike were significantly lower than in all other treatments (*p* < 0.05), resulting in yield reductions of 33.54% and 42.68% relative to TF and RF. When compared with TF and RF, the RF-P treatment exhibited no statistically significant differences in spike length or grain number per spike, although both parameters showed decreasing trends; consequently, its yield was 10.40% and 22.69% lower than that of TF and RF, respectively.

### 2.4. Effects of Different Fertilization Treatments on Nutrient-Use Dynamics

Relative to TF, RF significantly increased both the agronomic efficiency (AE) and partial factor productivity (PFP) of N, P, and K fertilizers. RF-K showed no significant differences in these indices for N and P compared with TF. In both RF-N and RF-P, all indices were significantly lower than in RF but remained comparable to TF. Specifically: RF-N reduced AE of P and K by 43.88% and 43.89%, and PFP of P and K by 41.60% and 27.90%, respectively. RF-P reduced AE of N and K by 27.71% and 27.27%, and PFP of N and K by 53.18% and 50.23%, respectively (Table 3).

### 2.5. Effects of Different Fertilization Treatments on Wheat Grain Quality

**Figure 4:**
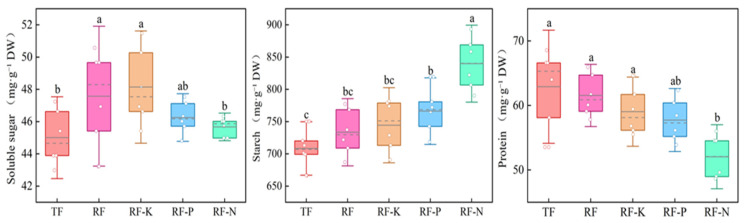
Effects of different fertilization treatments on wheat (*Triticum aestivum* L.) grain quality. Different letters indicate significant differences among treatments (*p* < 0.05).

Different fertilization treatments significantly affected wheat grain quality (Figure 4). Compared with TF, the RF and RF-K treatments significantly increased grain soluble sugar content, but had no significant effect on starch or protein content. The RF-N and RF-P treatments significantly increased starch content by 17.81% and 8.48%, respectively, relative to TF. Meanwhile, the RF-N treatment significantly reduced protein content (*p* < 0.05), with a decrease of 17.81%.

Grain nitrogen accumulation and protein yield were computed from grain yield and the corresponding nitrogen and protein concentrations (as detailed in Section 4.3.7). The RF treatment produced the highest grain nitrogen accumulation (233.99 kg ha^−1^) and protein yield (4444.63 kg ha^−1^), which were not statistically different from those recorded under TF (215.58 kg ha^−1^ and 3810.66 kg ha^−1^, respectively). Conversely, RF-N reduced both measures to 114.30 kg ha^−1^ and 1393.13 kg ha^−1^, corresponding to decreases of 46.98% and 63.44% relative to TF. Similarly, RF-P caused substantial reductions (149.06 kg ha^−1^ and 2296.05 kg ha^−1^), whereas RF-K (193.58 kg ha^−1^ and 3237.33 kg ha^−1^) exhibited no significant differences when compared with TF.

### 2.6. Effects of Different Fertilization Treatments on Nutrient Uptake and Harvest Indices

Nitrogen uptake efficiency did not differ significantly across fertilization treatments. Relative to TF, RF significantly increased both phosphorus and potassium uptake efficiency. In contrast, RF-N substantially reduced phosphorus and potassium uptake efficiency compared with TF and RF, with decreases of 24.00% and 38.71% for phosphorus, and 48.00% and 42.57% for potassium, respectively. Regarding nutrient harvest index, neither RF nor RF-K differed significantly from TF for N, P, or K indices. RF-N showed a significantly higher nitrogen harvest index, increasing by 18.64% relative to TF and 16.95% relative to RF. Additionally, RF-P exhibited a significantly higher potassium harvest index than TF. (Table 4).

### 2.7. Correlation Analysis of Factors Affecting Wheat Yield and Quality

**Figure 5:**
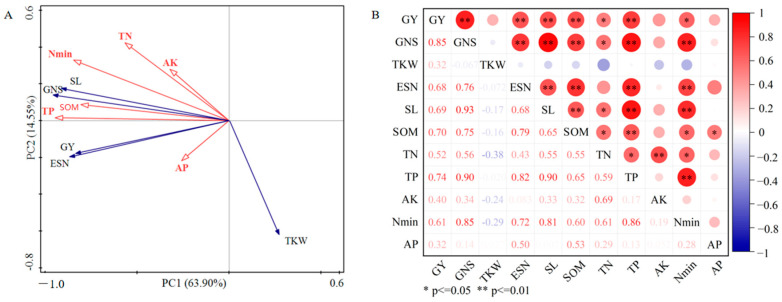
Correlation analysis of yield. (**A**) Redundancy analysis (RDA) of wheat yield and yield components driven by soil physicochemical properties; (**B**) Pearson correlation heatmap among yield components and soil nutrient indicators. GY, grain yield; GNS, grain number per spike; TKW, thousand-kernel weight; ESN, ear number pe hectare, SL, Spike length; SOM, soil organic matter; TN, total nitrogen; TP, total phosphorus; AK, available potassium; Nmin, mineral nitrogen; AP, available phosphorus.

Under the fertilization conditions in the Ningxia Yellow River Diversion Irrigation Area, significant correlations were observed between wheat grain yield and its components and soil nutrients. Redundancy analysis (RDA) showed that the first (63.90%) and second (14.55%) axis explained 78.45% of the total variation, indicating that soil environmental factors could effectively explain the variation in wheat yield and its components (Figure 5A). Forward selection by Monte Carlo Permutation tests further demonstrated that soil total phosphorus was the environmental factor most strongly associated with changes in wheat yield and its components (*p* = 0.002). Correlation analysis indicated that soil total phosphorus was not only significantly positively correlated with grain yield (*p* < 0.05), but also highly significantly positively correlated with grain number per spike, spike number per hectare, and spike length (*p* < 0.001). Soil organic matter and mineral nitrogen also showed similar positive correlations with these indicators (Figure 5B).

### 2.8. Correlation Analysis of Factors Affecting Wheat Quality

**Figure 6:**
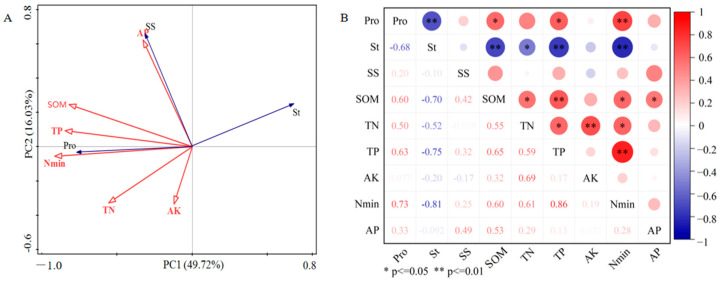
Correlation analysis of quality. (**A**) Redundancy analysis (RDA) of wheat quality parameters driven by soil physicochemical properties; (**B**) Pearson correlation heatmap among quality parameters and soil nutrient indicators. Pro, protein; ST, starch; SS, soluble sugar; SOM, soil organic matter; TN, total nitrogen; TP, total phosphorus; AK, available potassium; Nmin, mineral nitrogen; AP, available phosphorus.

Redundancy analysis (RDA) showed that the first and second axes explained 63.90% and 14.55% of the variation in wheat quality driven by soil physicochemical properties, respectively (Figure 6A). The forward selection procedure of the Monte Carlo test identified soil mineral nitrogen as the environmental factor most strongly associated with wheat quality components (*p* = 0.002). Correlation analysis further confirmed that soil mineral nitrogen content had a highly significant positive correlation with protein content (*p* < 0.001) and a highly significant negative correlation with starch content (*p* < 0.001). Soil organic matter and total phosphorus also showed a significant positive correlation with protein content (*p* < 0.05) and a significant negative correlation with starch content (*p* < 0.05). The correlations between soluble sugar content and all soil nutrient indicators were not significant (Figure 6B).

## 3. Discussion

### 3.1. Nitrogen Is a Key Factor Critical to the Synergistic Improvement of Wheat Yield and Quality in Ningxia’s Irrigated Silty Soil

The findings from the different fertilization treatments reveal that nitrogen serves as a key limiting factor for the synergistic improvement of both yield and quality of wheat in the irrigated silty soil of Ningxia. The negative effects of nitrogen deficiency (RF-N) on yield and quality were significantly greater than those of phosphorus and potassium deficiencies, which is consistent with most previous studies [12,13]. In the specific medium of Ningxia irrigated silty soil, the mineralization rate of soil organic nitrogen is slow under arid climatic conditions, resulting in a weak inherent nitrogen supply capacity [14]. Meanwhile, the high pH environment may exacerbate ammonia volatilization loss of nitrogen fertilizers (especially ammonium nitrogen), as reported in previous studies [15], This likely mechanism, together with the slow mineralization of soil organic nitrogen under arid conditions, collectively contributes to an “innate deficiency” of soil nitrogen availability. This observation further underscores the crucial limiting role of nitrogen within the local ecosystem. Our findings show that balanced application of nitrogen, phosphorus, and potassium allows a moderate reduction in fertilizer inputs to maintain stable yield and quality through nutrient synergy. In contrast, nitrogen deficiency causes a marked decline in both yield and quality. Sufficient nitrogen supply was associated with spike number per unit area, dry matter accumulation after anthesis, and the remobilization of stored reserves from vegetative organs to grains. By contrast, nitrogen deficiency causes substantial declines in vegetative growth parameters—including plant height, stem diameter, and leaf area—and ultimately leads to a significant drop in grain yield by severely restricting key yield components such as spike length and grain number per spike [16,17,18].

Among all treatments, nitrogen reduction caused the strongest suppression of wheat photosynthesis, resulting in a marked decline in the net photosynthetic rate (Pn) of flag leaves during the grain-filling stage (BBCH 7). This decline was accompanied by an abnormal elevation of intercellular CO_2_ concentration (Ci), suggesting a possible link to impaired photosynthetic biochemical capacity of mesophyll cells (non-stomatal limitation) [19]. Nevertheless, because chlorophyll concentration, SPAD index, and leaf nitrogen concentration were not determined in the present study, any interpretation of the mechanisms underlying photosynthetic limitation should be made with due caution. Redundancy analysis further highlighted soil mineral nitrogen content as the environmental factor most tightly correlated with variations in wheat grain quality. Consequently, providing an appropriate amount of nitrogen is not only critical for maintaining high photosynthetic efficiency and achieving high yield, but also a key factor for improving grain quality.

Under the RF-N treatment, soil organic matter (SOM) declined sharply within a single growing season. Three interconnected mechanisms may explain this change. First, nitrogen deficiency suppressed crop growth—reducing plant height, stem diameter, and leaf area—which decreased root biomass and rhizodeposition (the main source of fresh organic carbon), thereby limiting SOM formation [20]. Second, under N-limited conditions, soil microbes may adopt an “N-mining” strategy, accelerating the decomposition of existing SOM to acquire nitrogen; this process is well-recognized in N-limited ecosystems [21]. Third, reduced crop growth weakened the physical protection of SOM by root networks and reduced stable macroaggregate formation, making SOM more vulnerable to microbial decomposition [22]. Although the single-season SOM loss is substantial, similar rapid declines have been reported in other N-limited agroecosystems, particularly in coarse-textured soils with low initial SOM content [23]. Thus, this response should be interpreted cautiously, and long-term field studies are needed to determine whether this downward trend persists or stabilizes over time.

### 3.2. “Nitrogen-Phosphorus Synergy” as the Key Mechanism to Overcome the “Abundant Reserve but Low Efficiency” Paradox in Irrigated Silty Soil and Optimize Yield Structure

In contrast to nitrogen and phosphorus deficiencies, the omission of potassium (RF-K) did not result in a significant decrease in wheat yield. This absence of a yield response can be attributed to the high indigenous potassium supply of the irrigated silty soil (initial available K: 143 mg kg^−1^), which was sufficient to satisfy crop K requirements. Although nitrogen plays a direct role in driving grain quality formation [24], this study further reveals that, under the conditions of Ningxia irrigated silty soil, the effective supply of phosphorus fertilizer is a critical bottleneck limiting both yield improvement and nitrogen utilization efficiency, which is consistent with previous studies [25]. The recommended fertilization (RF) treatment did not achieve a significant yield breakthrough compared with the conventional fertilization (TF) treatment, whereas the phosphorus deficiency (RF-P) treatment led to a significant yield reduction. This indicates that insufficient phosphorus supply in this soil constrains overall production potential. This bottleneck primarily stems from the strong phosphorus fixation capacity of the irrigated silty soil, which may inhibit early root development and nitrogen uptake, thereby weakening the yield-increasing effect of nitrogen fertilizer [26]. Consequently, a marked discrepancy exists between the abundant nutrient reserves in the soil and the low crop nutrient uptake and utilization efficiency.

The RF treatment in this study successfully addressed this paradox through a synergistic optimization strategy with reduced nitrogen and phosphorus inputs. While lowering total nutrient input, the RF treatment maintained a continuous and stable supply of soil nutrients through a rationally optimized ratio, thereby supporting an ideal yield structure. Redundancy analysis further confirmed that soil total phosphorus content was the environmental factor most strongly associated with changes in wheat yield, and grain number per spike, spike number per hectare, and spike length were all highly significantly positively correlated with soil total phosphorus. In summary, through synergistic effects, the recommended fertilization treatment overcomes the phosphorus supply limitation while fully leveraging the yield-increasing effect of nitrogen, thereby promoting the synergistic optimization of photosynthetic production and grain accumulation. Ultimately, under reduced fertilization conditions, it achieves simultaneous improvements in spike number, grain number per spike, and grain weight, leading to higher yield. Compared to available phosphorus (AP), soil total phosphorus (TP) serves as a superior indicator of yield variability, owing to the pronounced phosphorus fixation characteristic of Ningxia’s irrigated silty soil, in which the majority of added phosphorus is quickly transformed into insoluble calcium-bound compounds [27]. Wheat plants are able to access this fixed phosphorus, rendering TP a dependable measure of the soil’s long-term phosphorus-supplying capability, whereas AP merely reflects a short-lived and readily extractable pool. TP demonstrates strong positive associations with spike number, grains per spike, and spike length, and emerges as the primary explanatory variable in redundancy analysis, highlighting its strong linkage to the overall phosphorus nutritional status that sustains yield across the entire growing season. Taken together, reduced fertilization in irrigated silty soil operates mainly via the synergistic interaction of nitrogen and phosphorus inputs, thereby overcoming the “high reserve but low efficiency” dilemma induced by phosphorus fixation and releasing the yield-enhancing potential of nitrogen. The underlying mechanisms of this synergy are rooted in plant physiological processes. Phosphorus enhances nitrogen uptake through multiple mechanisms. First, phosphorus promotes root growth—increasing root length, lateral branching, and root hair density—thereby expanding soil exploration and improving nitrate acquisition [28]. Second, as an essential component of ATP, phosphorus is essential for energy-dependent nitrogen assimilation. Phosphorus limitation reduces ATP availability, suppressing nitrate reduction and amino acid synthesis, which ultimately lowers nitrogen use efficiency [29]. Third, phosphorus deficiency downregulates nitrate and ammonium transporters, thereby impairing nitrogen uptake [30]. Together, these mechanisms explain why phosphorus availability strongly constrains nitrogen utilization. In our study, phosphorus deficiency (RF-P) reduced grain yield and nitrogen accumulation, whereas recommended fertilization (RF) sustained root proliferation, ATP-dependent nitrogen assimilation, and overall productivity through balanced nitrogen and phosphorus supply. Consistent with these physiological insights, the relationship between soil fertility and crop yield is complex and strongly shaped by fertilizer management. Appropriate nutrient management enhances crop nutrient uptake and yield by stimulating root growth and improving nutrient transport efficiency [31]. Yuan et al. (2024) reported that optimized fertilization boosts crop productivity by increasing soil fertility and nutrient uptake capacity [32]. Similarly, El-Sharkawy et al. (2024) provided evidence that optimized fertilization sustains soil fertility and crop productivity in intensive cereal systems, thereby strengthening mechanistic interpretations of nutrient-use efficiency [33]. Collectively, these findings indicate that the recommended fertilization (RF) strategy in our study increased yield not merely through higher soil total phosphorus, but also through improved nutrient availability and uptake efficiency—consistent with the principles of balanced fertilization.

### 3.3. Pathways to Synergistically Enhance Yield and Quality Through Soil–Plant System Regulation

Optimized fertilization can achieve synergistic improvement of wheat yield and quality [34]. In this study, the recommended fertilization maintained grain yield and quality at levels comparable to those of conventional fertilization, whereas nitrogen and phosphorus deficiencies led to simultaneous reductions in both yield and quality. Although conventional fertilization can achieve high yield and quality, it relies on high fertilizer input with substantial environmental costs [35]. This finding aligns with studies reporting that nutrient synergy achieves win-win outcomes for yield and quality in wheat production [36]. Redundancy analysis showed that soil total phosphorus was the key factor most strongly associated with yield formation, synchronously increasing spike number, grain number per spike, and spike length, which is consistent with the “key regulatory role of soil phosphorus pool in wheat yield components” [37]. Soil mineral nitrogen was similarly identified as the factor most strongly associated with grain quality, showing a highly significant positive correlation with protein accumulation. This association is consistent with the understanding that nitrogen supply plays an important role in grain protein formation [38]. The strong association between mineral nitrogen and grain protein content observed in this study is also consistent with previous reports demonstrating the importance of available soil nitrogen pools for crop nutrition and plant performance [39]. These soil nutrients regulate yield and quality primarily through plant physiological processes. On the one hand, adequate supply of total phosphorus was associated with high photosynthetic performance of flag leaves after anthesis, which corresponded to increased grain yield [40]. On the other hand, continuous mineral nitrogen supply after anthesis was associated with higher plant nitrogen accumulation and greater grain protein content, consistent with the role of post-anthesis nitrogen remobilization [41,42]. Grain protein formation depends on post-anthesis nitrogen remobilization and source–sink balance. During grainfilling, nitrogen stored in vegetative tissues is translocated to developing kernels via enzymes and transporters, supplying 50–90% of mature grain nitrogen [43]. Nitrogen availability affects grain quality through two pathways: preserving flag leaf photosynthesis to enhance source strength, and upregulating storage protein genes to enhance sink strength [44]. In our study, recommended fertilization (RF) promoted efficient post-anthesis nitrogen transfer to grains, sustaining grain nitrogen accumulation and protein yield. In contrast, nitrogen deficiency (RF-N) disrupted this process, leading to substantial reductions in both parameters. Sufficient mineral nitrogen availability during the grain-filling stage is linked to increased protein and starch storage contents in grains, creating a dynamic equilibrium between protein and starch synthesis [45]. Higher soil total phosphorus supports efficient nitrogen assimilation by providing both energy and structural benefits through an optimized yield framework, and the combined effect of these two nutrients contributes to the enhancement of both yield and quality. The marked declines in grain nitrogen accumulation and protein yield observed under nitrogen and phosphorus deficiency further highlight the essential roles of these nutrients in both yield formation and grain quality. Consistent with these findings, RF significantly improved phosphorus and potassium uptake efficiency, while RF-N showed a higher nitrogen harvest index, indicating enhanced N remobilization to grains under N deficiency. These observations align with the correlation analysis, which identified soil mineral nitrogen as the primary factor governing grain protein content. Moreover, the similar performance of RF and TF in terms of grain N accumulation and protein yield suggests that the recommended fertilization strategy preserved grain quality while lowering nutrient inputs.

Coordinated improvement of both yield and quality depends on the simultaneous optimization of two key factors through fertilizer management: the phosphorus-driven foundation for yield and the nitrogen-associated regulation of quality metabolism. In the irrigated silty soil region of Ningxia, basal phosphorus application should be emphasized to support spike development. Alongside a well-balanced split application of nitrogen fertilizer (both basal and topdressed) to ensure adequate nitrogen availability during the grain-filling stage, these practices together establish a systematic management strategy described as “securing yield with phosphorus and precisely enhancing quality with nitrogen,” ultimately leading to the integration of high productivity, superior grain quality, and efficient resource use in wheat production. In the present study, the recommended fertilization (RF) treatment achieved higher partial factor productivity (PFP) and agronomic efficiency (AE) than conventional fertilization (TF), indicating that this approach enhances nutrient-use efficiency without reducing grain yield. Compared to TF, RF increased the PFP of nitrogen, phosphorus, and potassium by 48.8%, 49.6%, and 48.0%, respectively, and raised the AE of these nutrients by 94.7%, 188.0%, and 84.2%, respectively (Table 3). These findings strongly support the basal plus split-topdressing nitrogen management strategy used here: 60% applied as basal fertilizer and the remaining 40% topdressed in three equal splits at tillering, jointing, and heading stages. This strategy synchronizes nitrogen supply with crop demand, thereby minimizing nutrient losses and improving uptake efficiency [46]. Consistent with studies in arid agricultural systems, optimized water and nitrogen management can improve both wheat yield and water use efficiency [47]. Additionally, recovering resources from agricultural waste provides environmental and economic advantages that further support sustainable nutrient management [48]. In contrast, the much lower PFP and AE in RF-N and RF-P suggest that N and P deficiencies not only reduce yield but also severely limit nutrient-use efficiency, highlighting the need for balanced N and P supply in this irrigated silty soil.

This study was conducted during a single growing season (2022) at one experimental site. Therefore, the conclusions regarding nutrient limitation patterns (nitrogen as the primary limiting factor and phosphorus as a secondary limiting factor) are based on one-year field data. Given the potential inter-annual variability in climate, soil conditions, and crop performance, the findings should be interpreted with caution. Long-term, multi-site field experiments are needed to validate the observed nutrient limitation patterns across the entire Ningxia irrigated silty soil region.

## 4. Materials and Methods

### 4.1. Study Area

The experiment was conducted in March 2022 at Beicun Village, Wangtuan Town, Tongxin County, Ningxia (36°51′ N, 105°59′ E). This region has a typical temperate continental arid climate, classified as BSk (cold semi-arid) according to the Köppen–Geiger climate classification system, with an annual mean temperature of 8.7 °C and annual precipitation ranging from 170 to 200 mm. Per the WRB classification, the soil is a Calcaric Fluvisol. The soil is silty loam (irrigated silty soil). According to regional surveys of the Ningxia Yellow River Irrigation Area, the typical particle size distribution is approximately 35% sand, 48% silt, and 17% clay [49]. The basic physicochemical properties of the topsoil (0–20 cm) before the experiment were as follows: bulk density 1.08 g·cm^−3^, organic matter 16.25 g·kg^−1^, mineral nitrogen 32.01 mg·kg^−1^, available phosphorus 24.69 mg·kg^−1^, available potassium 143.01 mg·kg^−1^, total nitrogen 0.59 g·kg^−1^, total phosphorus 0.57 g·kg^−1^, and pH 7.83.

Under the World Reference Base (WRB) classification, the study site soil is designated as Calcaric Fluvisol. The upper 20 cm of the soil profile has a silty loam texture, consisting of 35% sand, 48% silt, and 17% clay. The cation exchange capacity (CEC) is 12.6 cmol^+^ kg^−1^, base saturation is 92.5%, and calcium carbonate (CaCO_3_) concentration ranges from 45 to 75 g kg^−1^ [5].

### 4.2. Experimental Design

This experiment was a field plot trial using a randomized complete block design with five treatments and three replications, resulting in a total of 15 plots. Each plot had an area of 164.43 m^2^ (8.7 m × 18.9 m). Ridges of 30 cm in height and 50 cm in width were built between plots. The treatments were as follows: (1) conventional farmer fertilization (TF): 270 kg N ha^−1^, 120 kg P_2_O_5_ ha^−1^, 75 kg K_2_O ha^−1^; (2) recommended fertilization (RF): 215 kg N ha^−1^, 95 kg P_2_O_5_ ha^−1^, 60 kg K_2_O ha^−1^; (3) nitrogen deficiency (RF-N): 95 kg P_2_O_5_ ha^−1^, 60 kg K_2_O ha^−1^; (4) phosphorus deficiency (RF-P): 215 kg N ha^−1^, 60 kg K_2_O ha^−1^; (5) potassium deficiency (RF-K): 215 kg N ha^−1^, 95 kg P_2_O_5_ ha^−1^. The recommended fertilization (RF) treatment was established using regional soil test data and local agricultural extension guidelines for wheat cultivation in the Ningxia Yellow River Diversion Irrigation Area.

The tested chemical fertilizers were urea (46% N), superphosphate (47% P_2_O_5_), and potassium chloride (60% K_2_O). All phosphorus and potassium fertilizers, together with 60% of the nitrogen fertilizer, were applied as basal fertilizer. The remaining 40% of the nitrogen fertilizer was topdressed in three splits: 50% at the tillering stage (April 10), 30% at the jointing stage (May 10), and 20% at the heading stage (May 30).

Prior to sowing, the experimental plots were plowed with a mouldboard plow to a depth of 25–30 cm, and then rototilled to create a well-structured seedbed. A border (flood) irrigation system was used, providing a total of four watering events during the wheat growth cycle: before sowing, at jointing, at heading, and during grain filling. Each irrigation delivered approximately 600 m^3^ ha^−1^ of water. Routine local practices were followed for weed and pest management.

The wheat variety Ningchun 4 was used as the test material, and conventional pest and disease control, irrigation, and cultivation management were applied. Before harvest, soil samples from the topsoil layer (20 cm) of each treatment were collected using a multi-point sampling method. The samples were taken back to the laboratory, air-dried naturally, and then ground to pass through 20-mesh and 100-mesh sieves, respectively, for subsequent analysis. Representative plants were selected from each plot, oven-dried at 105 °C for 0.5 h to deactivate enzymes, and then dried at 60 °C to constant weight. The samples were subsequently ground into fine powder and stored for further analysis.

### 4.3. Determination Indicators and Methods

#### 4.3.1. Soil Sample Collection and Analysis

Before wheat harvest, soil samples were collected from the plow layer (0–20 cm) of each treatment using a multi-point sampling method. After air-drying, grinding, and sieving, the following indicators were determined: soil total nitrogen (Kjeldahl method), organic matter (potassium dichromate oxidation-external heating method), total phosphorus (H_2_SO_4_-HClO_4_ digestion-molybdenum antimony colorimetric method), available phosphorus (Olsen method), and available potassium (NH_4_OAc extraction-flame photometry method) [50]. Soil mineral nitrogen was calculated as the sum of ammonium nitrogen and nitrate nitrogen, which were extracted with 1 mol L^−1^ KCl and then determined using a continuous flow injection analyzer (AMS Alliance Futura, Frepillon, France).

#### 4.3.2. Plant Sample Collection and Analysis

At the jointing(BBCH 3), heading(BBCH 5), grain-filling(BBCH 7), and harvest(BBCH 9) stages of wheat, six plants with uniform growth were selected from each plot, with three replications per treatment, for the following measurements: Wheat plant height: Measured from the base to the top of the plant using a tape measure. Wheat stem diameter: Measured using a vernier caliper with an accuracy of 0.01 mm. Wheat leaf area: Determined using a portable leaf area meter (YMJ-D, Top Instrument Co., Ltd., Hangzhou, China).

#### 4.3.3. Flag Leaf Photosynthetic Parameters

On a sunny and windless day at the grain-filling(BBCH 7) stage, six representative flag leaves were randomly selected from each treatment plot. The net photosynthetic rate (Pn), stomatal conductance (Gs), intercellular CO_2_ concentration (Ci), and transpiration rate (Tr) were measured using a Li-6400 portable photosynthesis system. (LI-COR Biosciences, Lincoln, NE, USA). All parameters were measured three times per sample.

#### 4.3.4. Yield Components and Plant Measurement

After wheat maturity, a 1 m double-row sample was collected from each plot for plant measurement. The spike number per hectare, spike length, grain number per spike, and 1000-grain weight were determined. The grains from each plot were harvested and weighed to measure the actual yield, which was then converted into grain yield per hectare.

#### 4.3.5. Wheat Grain Quality Analysis

The grain samples obtained from the plant measurement were analyzed for nutritional quality. Soluble sugar content was determined using the anthrone method. Starch content was measured by the dual-wavelength method to determine the contents of amylose and amylopectin, with total starch content calculated as the sum of amylose and amylopectin. Protein content was determined using the Kjeldahl method to measure nitrogen content, and the protein content was calculated as nitrogen content multiplied by 5.7.

#### 4.3.6. Nutrient-Use Efficiency Calculation

Partial factor productivity (PFP) of N, P, and K (kg kg^−1^) = Grain yield (kg ha^−1^)/Nutrient application rate (kg ha^−1^)

Agronomic efficiency (AE) of N, P, and K (kg kg^−1^) = (Yield in fertilized plot − Yield in deficient plot)/Nutrient application rate (kg ha^−1^)

#### 4.3.7. Grain Nitrogen Accumulation and Protein Yield Calculation

Grain nitrogen accumulation (kg ha^−1^) = Grain yield (kg ha^−1^) × Grain N content (%)/100

Protein yield (kg ha^−1^) = Grain yield (kg ha^−1^) × Grain protein content (%)/100

#### 4.3.8. Nutrient Uptake Efficiency and Harvest Index Calculation

Nutrient uptake efficiency (NUpE) for N, P, and K was calculated as:

Nitrogen uptake efficiency (NUpE) of N, P, and K (%) = Total N uptake (kg ha^−1^)/N application rate (kg ha^−1^)

Nutrient harvest index (NHI) for N, P, and K was calculated as:

Nutrient harvest index (NHI) of N, P, and K = Nutrient accumulation in grains (kg ha^−1^)/Total aboveground nutrient accumulation (kg ha^−1^)

### 4.4. Data Processing

Data were organized using Microsoft Excel 2010 software (Microsoft Corp., Redmond, WA, USA). One-way analysis of variance (ANOVA) was performed using SPSS 26.0 software (IBM Corp., Armonk, NY, USA), and significant differences among treatments were determined by Duncan’s new multiple range test (*p* < 0.05). Prior to ANOVA, the normality (Shapiro–Wilk test) and homogeneity of variances (Levene’s test) were checked, and all indicators met the assumptions for ANOVA. Plant growth indicators (plant height, stem diameter, leaf area) measured at different growth stages (jointing, heading, filling, and harvest) were analyzed separately for each time point using independent one-way ANOVA. Redundancy analysis was conducted using Canoco 5.0 (Microcomputer Power, Ithaca, NY, USA) based on plot-level data (i.e., each plot was treated as an independent sample, *n* = 3). Graphs were plotted using Origin 2021 software (OriginLab Corp., Northampton, MA, USA). Pearson correlation analysis was used to analyze the correlations among yield, quality indicators, soil properties, and plant traits.

## 5. Conclusions

By comparing the effects of different fertilization patterns on wheat in the Ningxia Yellow River Diversion Irrigation Area, the following conclusions are drawn:

(1) In this region, nitrogen availability is the main constraint on wheat productivity. Reducing nitrogen input strongly suppresses photosynthesis after anthesis, resulting in fewer spikes, fewer grains per spike, lower grain protein content, and decreased soil mineral nitrogen and organic matter levels. Soil mineral nitrogen serves as the primary environmental factor strongly associated with grain protein formation.

(2) Phosphorus is a secondary limiting factor and exhibits a significant synergistic effect with nitrogen. Soil total phosphorus is significantly positively correlated with yield and is the factor most strongly associated with yield increase. Adequate phosphorus supply plays a key role in ensuring nitrogen fertilizer efficiency and stabilizing grain number per spike.

(3) The recommended fertilization treatment, by maintaining high post-anthesis photosynthetic performance and plant nitrogen uptake, can achieve synergistic maintenance of yield and protein content, making it suitable for green and high-yield production in this region.

In summary, this study proposes a provisional nutrient limitation framework for the study area’s irrigated silty soil, where wheat production exhibits “nitrogen predominance with phosphorus complementarity.” However, because the experiment was limited to a single site, one growing season, and one wheat variety (Ningchun 4), broader validation through long-term, multi-site, and multi-cultivar studies is required to confirm the general applicability of this pattern across the Ningxia irrigated silty soil region.

## Figures and Tables

**Table 1:** Changes in soil nutrients under different fertilization treatments.

Treatment	AK (mg·kg^−1^)	AP (mg·kg^−1^)	Nmin (mg·kg^−1^)	TN (g·kg^−1^)	TP (g·kg^−1^)	SOM (g·kg^−1^)
TF	138.93 ± 5.27 ab	13.59 ± 1.07 ab	18.14 ± 1.36 a	1.06 ± 0.04 ab	0.66 ± 0.06 ab	12.70 ± 0.40 ab
RF	149.34 ± 8.22 a	15.32 ± 1.17 a	17.32 ± 0.54 ab	1.09 ± 0.02 a	0.72 ± 0.02 b	14.54 ± 0.47 a
RF-K	128.06 ± 2.65 b	13.66 ± 0.68 ab	16.59 ± 0.79 ab	1.02 ± 0.05 a	0.62 ± 0.06 b	12.89 ± 0.85 b
RF-P	142.22 ± 14.32 ab	12.61 ± 1.16 b	14.34 ± 1.12 c	1.00 ± 0.02 b	0.66 ± 0.04 ab	11.75 ± 0.97 c
RF-N	136.96 ± 4.92 ab	13.66 ± 0.20 ab	13.18 ± 1.41 c	0.86 ± 0.05 c	0.60 ± 0.02 b	9.54 ± 1.21 d

Values are presented as mean ± standard deviation. Different lowercase letters in the same column indicate significant differences (*p* < 0.05). SOM, soil organic matter; TN, total nitrogen; TP, total phosphorus; AK, available potassium; Nmin, mineral nitrogen; AP, available phosphorus.

**Table 2:** Effects of different fertilization treatments on wheat grain yield and yield components.

Treatment	Number of Hectare Spikes (10^4^·hm^−2^)	Spike Length (cm)	Number of Grains	Thousand Grain Weight (g)	Grain Yield (kg·hm^−2^)
TF	599.21 ± 17.27 a	8.97 ± 0.48 a	34 ± 2.26 a	29.80 ± 4.19 a	3427 ± 205 a
RF	611.70 ± 32.05 a	8.68 ± 0.55 a	35 ± 0.63 a	31.56 ± 5.00 a	3857 ± 297 a
RF-K	607.31 ± 21.76 a	8.32 ± 0.61 a	31 ± 2.78 a	30.99 ± 5.08 a	3194 ± 386 ab
RF-P	586.72 ± 25.94 a	8.28 ± 0.56 a	30 ± 3.75 a	31.72 ± 6.44 a	2582 ± 467 b
RF-N	579.88 ± 26.40 a	6.03 ± 0.52 b	18 ± 1.87 b	31.75 ± 2.00 a	2211 ± 58 c

Values are presented as mean ± standard deviation. Different lowercase letters in the same column indicate significant differences (*p* < 0.05).

**Table 3:** Effects of different fertilization treatments on nutrient-use efficiency.

Treatment	PFP of N (kg kg^−1^)	PFP of P (kg kg^−1^)	PFP of K (kg kg^−1^)	AE of N (kg kg^−1^)	AE of P (kg kg^−1^)	AE of K (kg kg^−1^)
TF	12.32 ± 1.76 b	27.73 ± 1.71 bc	44.37 ± 2.73 bc	4.13 ± 0.55 b	3.99 ± 2.36 b	8.29 ± 4.71 b
RF	18.33 ± 2.06 a	41.48 ± 4.65 a	65.67 ± 7.37 a	8.04 ± 2.19 a	11.49 ± 3.80 a	15.27 ± 2.57 a
RF-K	14.06 ± 2.35 b	31.83 ± 5.31 b	/	3.78 ± 2.54 b	4.71 ± 0.38 b	/
RF-P	13.25 ± 1.64 b	/	47.48 ± 5.87 b	2.96 ± 1.54 b	/	7.46 ± 0.60 b
RF-N	/	23.28 ± 0.61 c	36.85 ± 0.97 c	/	6.71 ± 3.48 b	11.01 ± 0.48 a

Values are presented as mean ± standard deviation. Different lowercase letters in the same column indicate significant differences (*p* < 0.05). PFP, Partial factor productivity; AE, Agronomic efficiency.

**Table 4:** Effects of different fertilization treatments on nutrient uptake and harvest indice.

Treatment	NUpE-N	PUpE-P	KUpE-K	NHI-N	PHI-P	NHI-K
TF	0.84 ± 0.05 a	0.25 ± 0.01 b	1.95 ± 0.13 b	0.48 ± 0.06 b	0.48 ± 0.09 a	0.14 ± 0.02 b
RF	0.74 ± 0.06 a	0.31 ± 0.02 a	2.56 ± 0.14 a	0.49 ± 0.02 b	0.52 ± 0.05 a	0.17 ± 0.01 ab
RF-K	0.92 ± 0.05 a	0.28 ± 0.02 ab	/	0.51 ± 0.02 ab	0.48 ± 0.08 a	0.18 ± 0.03 ab
RF-P	0.85 ± 0.18 a	/	1.9 ± 0.15 b	0.52 ± 0.05 ab	0.56 ± 0.03 a	0.20 ± 0.01 a
RF-N	/	0.19 ± 0.02 c	1.47 ± 0.15 c	0.59 ± 0.06 a	0.54 ± 0.09 a	0.16 ± 0.04 ab

Values are presented as mean ± standard deviation. Different lowercase letters in the same column indicate significant differences (*p* < 0.05). NUpE, Nutrient uptake efficiency; NHI, Nutrient harvest index.

## Data Availability

The original contributions presented in this study are included in the article. Further inquiries can be directed to the corresponding author.
